# 4045 A TL1 Team Approach to Identify Factors Affecting Rural Tobacco Users’ Participation in Research and Quitting Tobacco Use

**DOI:** 10.1017/cts.2020.352

**Published:** 2020-07-29

**Authors:** Neo Gebru, Rachel Elisabeth Damiani, Janice Krieger, Robert F Leeman

**Affiliations:** 1 University of Florida Clinical and Translational Science Institute

## Abstract

OBJECTIVES/GOALS: Guided by the health belief model and social identity theory, we aim to identify socio-cultural and psychological factors that influence rural tobacco users a) participation in research and b) quitting tobacco use. We also explore how citizen scientists are perceived as disseminators of messages. METHODS/STUDY POPULATION: In Phase I of this multi-stage project, we are conducting in-depth interviews with approximately 30 tobacco users. Interviews are on-going, and have been conducted with 16 participants thus far from four rural counties in Florida. The interview consists of semi-structured questions and multiple validated questionnaires. Specifically, we ask a series of questions about participants’ barriers to participating in research, tobacco use history, and internet use and message preferences. Additionally, we include questionnaires on participants’ substance use, nicotine dependence, motivation to quit, and willingness to participate in research studies. RESULTS/ANTICIPATED RESULTS: Initial findings suggest that rural tobacco users have an overall positive perception of research, and many choose to participate in research for altruistic reasons (i.e. they want to help others). Further, participants noted described feeling stigmatized due to their tobacco use. Although most began smoking to fit in with their community, many now feel on the outs. Participants also reported logistical barriers to participating in research, including lack of transportation. DISCUSSION/SIGNIFICANCE OF IMPACT: Findings can inform the development of recruitment materials to resonate with rural adults, including by emphasizing the collective potential to help by participating. This interdisciplinary highlights areas for collaboration to enhance the reach of health education and public health messages.

